# Recent Patents of Interest to Dentists

**Published:** 1906-11

**Authors:** 


### RECENT PATENTS OF INTEREST TO DENTISTS.

827,824, to Frederick W. Stephan. Dental articulator.

827,910, to John A. Hollenberger. Apparatus for administering liquid anesthetics.

827,965, to George Engle. Tooth-brush.

828,109, to Adolph Grunstein. Apparatus for stamping and shaping dental metal crowns.

828, 393, to Homer Emerson. Mirror for brushes or the like.

829,119, to Frank X. Mellen. Dental plugger.

829,395, to Nathan K. Garhart, Jr. Dental engine.

Copies of the above patents may be obtained for ten cents each by addressing John A. Saul, Solicitor of Patents, Fendall Building, Washington, D. C.

Copies of the above patents may be obtained for ten cents each by addressing John A. Saul, Solicitor of Patents, Fendall Building, Washington, D. C.

